# Physiological, Transcriptional, and Metabolic Responses Associated with Pyrophosphate-Mediated Alleviation of Cadmium Stress in Arabidopsis

**DOI:** 10.3390/plants15182864

**Published:** 2026-09-19

**Authors:** Yang Yang, Lu Wang, Pengpeng He, Jin Wang, Chunqiao Wang, Chao Wang, Ziwei Jiao

**Affiliations:** 1 College of Biological Sciences and Technology, Yili Normal University, Yining 835000, China; 2 Xinjiang Key Laboratory of Lavender Resource Protection and Utilization, Yili Normal University, Yining 835000, China; 3 College of Landscape and Ecological Engineering, Hebei University of Engineering, Handan 056038, China

**Keywords:** chemical remediation, phytotoxicity, phosphate amendment, nitrogen assimilation, metabolite profiling

## Abstract

Pyrophosphate serves as a vital reagent for the chemical remediation of soil heavy metal contamination. However, reports regarding how pyrophosphate regulates plant cadmium (Cd) tolerance at the physiological level remain scarce. In this study, we combined phenotypic, physiological, transcriptomic, and metabolomic approaches to comprehensively elucidate the response of Arabidopsis to Cd stress mediated by sodium pyrophosphate (Na-PPi). The results demonstrated that oxidative stress triggered by Cd significantly suppressed the growth of Arabidopsis seedlings. In contrast, exogenous Na-PPi effectively lowered the contents of MDA, H_2_O_2_, and O_2_^•−^, and concurrently diminished Cd accumulation in the plants under the Cd stress condition. Meanwhile, Na-PPi significantly altered the expression of genes associated with “phenylpropanoid biosynthesis” and “cell wall biosynthesis”. Furthermore, Na-PPi application under Cd stress significantly enhanced the expression of the nitrate transporter genes *NRT2.1* and *NRT2.6* and promoted nitrogen accumulation in Arabidopsis plants. Metabolomic analysis revealed that Na-PPi significantly altered the metabolic profile of Arabidopsis under Cd stress, increasing the accumulation of polydatin while decreasing the levels of nicotinic acid, baicalin, camalexin, and glutathione. These findings provide a crucial scientific basis for a deeper understanding of pyrophosphate-mediated heavy metal soil remediation from a plant-level perspective.

## 1. Introduction

Rapid industrialization and urbanization have led to increasing releases of Cd into the environment through multiple pathways, including irrigation with contaminated water, the application of Cd-containing agricultural amendments, and emissions from metallurgical activities, making Cd one of the most concerning environmental pollutants worldwide [1,2]. Due to its persistence and high bioavailability in ecosystems, excessive Cd exposure exerts pronounced phytotoxic effects, including disruption of cellular membrane integrity and function, interference with the uptake and translocation of essential mineral nutrients, induction of oxidative stress via reactive oxygen species (ROS) accumulation, and suppression of photosynthesis and nutrient assimilation [3,4]. Collectively, these adverse effects reduce crop yield and quality, posing a serious threat to food security [5,6]. Furthermore, Cd exhibits strong bioaccumulation potential and undergoes trophic magnification through the food chain, ultimately entering the human body via dietary intake and posing serious health risks, including renal dysfunction, bone metabolism disorders, and potential carcinogenicity [7]. Therefore, a comprehensive understanding of Cd behavior and transformation in soil–plant systems, together with the development of efficient and sustainable remediation strategies, is essential for protecting ecosystem health and safeguarding public health.

Chemical remediation is one of the most widely used approaches for managing Cd-contaminated soils. It involves the application of chemical agents to alter the chemical speciation, mobility, and bioavailability of Cd, thereby reducing its ecological risks and phytotoxicity [8,9,10]. Due to its relatively simple implementation and rapid remediation performance, chemical remediation has been widely used for moderately to heavily Cd-contaminated agricultural soils and industrial sites [11]. Recent studies further illustrate its efficacy through diverse agent-based interventions. For instance, Dong et al. reported that chelating agents, such as ethylenediaminetetraacetic acid, citric acid, and tetrasodium glutamate diacetate, promoted the transformation of Cd and lead (Pb) toward the residual fraction in soil, while decreasing their proportions in the weak acid-extractable and reducible states, thereby alleviating their adverse impacts on soil ecology [12]. Furthermore, incubation with sulfidated nanoscale zero-valent iron (S-nZVI) reduced the exchangeable fraction of Cd by more than 97.6%, concomitantly transforming it into less bioavailable forms, primarily associated with iron-manganese oxides and organic matter [13]. Meanwhile, Xia et al. demonstrated that soil washing with carboxyalkylthiosuccinic acid (CETSA) and a maleic acid–acrylic acid copolymer (MA/AA), both of which exhibit excellent water solubility and biodegradability, effectively reduced the environmental risks associated with Cd, Pb, and Zn contamination without significantly altering soil physicochemical properties [14].

Additionally, phosphate compounds are regarded as effective agents for modulating the mobility of heavy metals in soil, primarily through their strong adsorption capacity and ability to induce precipitation [15,16,17,18]. For instance, the co-application of sepiolite with two phosphate amendments (calcium magnesium phosphate and calcium superphosphate) during the remediation of Cd-contaminated soil significantly reduced Cd concentrations in brown rice by decreasing both soil HCl-extractable and DTPA-extractable Cd fractions [17]. Furthermore, the application of monoammonium phosphate at 0.8% (*w*/*w*) reduced Cd concentrations in wheat grains and rice [19]. Tetrasodium pyrophosphate (Na_4_P_2_O_7_; Na-PPi) has been reported as an efficient and biodegradable chemical agent for removing metals from the precipitated, exchangeable, and organic fractions in soils [20,21]. It can also react with Cd to form Cd pyrophosphate, which represents the predominant Cd-phosphorus precipitate species [22,23]. In plant cells, inorganic pyrophosphate (PPi) is generated as a byproduct of biosynthetic processes involving proteins, lipids, nucleic acids, and polysaccharides, and plays a critical role in stress responses and energy metabolism [24,25,26]. Although pyrophosphate-based remediation strategies for heavy metal-contaminated soils have demonstrated promising performance, their roles in regulating plant physiological and molecular responses remain poorly understood.

In this study, we systematically investigated the physiological regulatory roles of Na-PPi in Arabidopsis under Cd stress. Specifically, we evaluated key physiological parameters, including plant growth, reactive oxygen species (ROS) accumulation, antioxidant enzyme activities, and metal content, to assess how PPi modulates plant stress tolerance. Furthermore, we performed transcriptomic and metabolomic profiling to comprehensively characterize the molecular and biochemical reprogramming induced by Na-PPi during Cd exposure. By combining these multi-omics approaches with physiological data, this study provides a comprehensive understanding of the physiological and molecular responses of plants to PPi-assisted Cd remediation. This work provides new insights into the biological functions of PPi beyond its established role in cellular metabolism and provides a foundation for identifying candidate genetic and metabolic regulators that could be harnessed to enhance Cd tolerance in plants.

## 2. Materials and Methods

### 2.1. Plant Growth and Cd Treatment

The wild-type *Arabidopsis thaliana* Columbia-0 (Col-0) ecotype was selected for all experiments. Seeds were surface-sterilized with 75% ethanol (1 min) followed by sodium hypochlorite (1% ClO^−^; 10 min), and then stratified at 4 °C in the dark for 48 h. Seeds were then transferred to 1/2-strength Murashige and Skoog (MS) medium containing 50 and 75 μM of CdCl_2_, either in the presence or absence of 1 mM Na-PPi. Given that Na-PPi could affect the pH of 1/2 MS medium, the Na-PPi-free 1/2 MS medium was adjusted to pH 7.3 with 1 M NaOH, as previously described [24]. After emergence, seedlings remained in a growth chamber for a further 9 d under 22 °C, a 16 h light/8 h dark cycle, 60% relative humidity, and a photon flux density of 75 μmol m^−2^·s^−1^.

A pot experiment was conducted using *Lavandula angustifolia*. Seeds were surface-sterilized as described for Arabidopsis and evenly sown on the surface of moist humus soil. After germination and growth for 8 d, two vigorous and uniform seedlings were transplanted into each pot filled with humus soil. The pots measured 9 cm in outer diameter, 8 cm in inner diameter, 7.9 cm in height, and had a volume of 0.35 L, with matching saucers (10.8 cm in diameter). During this period, each pot was irrigated weekly with 50 mL of 1/2 Hoagland solution [27]. Greenhouse conditions were maintained at 24–25 °C, 50–60% relative humidity, a 16 h light/8 h dark photoperiod, and a light intensity of 80–90 μmol m^−2^·s^−1^. After 20 d of growth, plants were subjected to four treatments using 1/2 Hoagland nutrient solution as the basal medium: (1) 1/2 Hoagland solution (control), (2) 1 mmol·L^−1^ Na-PPi, (3) 50 μmol·L^−1^ CdCl_2_, and (4) 50 μmol·L^−1^ CdCl_2_ + 1 mmol·L^−1^ Na-PPi. For the Na-PPi-free treatments, the pH was adjusted to 7.3 as described above. During the treatment period, 50 mL of the corresponding treatment solution was added weekly to the saucer of each pot for a total of 80 d. At the end of the experiment, plants were carefully removed from the soil, and the roots were rinsed thoroughly with tap water before photographing and determining the fresh weight of each plant.

### 2.2. Determination of Metal Concentrations, Nitrogen Content, and Cell Wall Components

Metal concentrations were determined according to the method described by Lei et al. [28] and Gong et al. [29]. Briefly, plant samples were harvested, gently rinsed with distilled water, and blotted dry with filter paper to remove surface moisture. A 0.50 g aliquot of fresh tissue was then transferred to a digestion vessel and treated with 10 mL of HNO_3_/H_2_O_2_ (4:1, *v*/*v*). Digestion was performed at 160 °C for 1 h. After cooling, the inner walls of the tubes were rinsed thoroughly, and the digests were quantitatively transferred to 50 mL volumetric flasks and diluted to volume with ddH_2_O. Metal concentrations in the digests were determined using a flame atomic absorption spectrophotometer (M6 AA System, Thermo Fisher Scientific, Waltham, MA, USA).

For the determination of N content, the dried tissues were finely ground and digested with concentrated H_2_SO_4_ using a catalyst mixture of K_2_SO_4_, CuSO_4_, and Se (100:10:1, *w*/*w*/*w*). Total nitrogen content was determined by the sodium salicylate–sodium dichloroisocyanurate colorimetric method following the procedure described by Wang et al. [30].

The content of covalently soluble pectin (CSP) was determined using a kit (Cat. No. G0706F) purchased from Suzhou Grace Biotechnology Co., Ltd. (Suzhou, China). The contents of ionic-soluble pectin (ISP), cellulose, and hemicellulose were measured using corresponding kits (Cat. Nos. BC4150, BC4280, and BC4440, respectively) obtained from Beijing Solarbio Science & Technology Co., Ltd. (Beijing, China). All procedures were performed strictly in accordance with the manufacturer’s instructions.

### 2.3. Determination of MDA, H_2_O_2_, and O_2_^•−^ Contents and Antioxidant Enzyme Activities

Malondialdehyde (MDA), an indicator of lipid peroxidation, was assayed using the thiobarbituric acid (TBA) reaction using the method of Gong et al. [29]. Briefly, 0.3 g of fresh seedlings was homogenized in 2 mL of 10% trichloroacetic acid (TCA) solution on ice, followed by centrifugation at 5000 *g* for 10 min to obtain the extract. The resulting supernatant was mixed with an equal volume of 0.3% TBA and heated in a boiling water bath for 40 min. After cooling and centrifugation (5000 *g*, 10 min), the absorbance was measured, and MDA concentration was calculated using the following equation: C(MDA) = 6.45 × (A535 − A600) − 0.56 × A440. H_2_O_2_ content was determined using the potassium iodide (KI) method by measuring the absorbance at 390 nm [31]. Superoxide anion (O_2_^•−^) content was determined using the hydroxylamine method [32].

Fresh Arabidopsis seedlings (0.5 g) were homogenized in 50 mM PBS (pH 7.8) supplemented with 1 mM EDTA and 1% (*w/v*) PVP. After centrifugation at 15,000× *g* for 10 min at 4 °C, the supernatant was collected for protein quantification using the Bradford assay and subsequent determination of superoxide dismutase (SOD), catalase (CAT), peroxidase (POD), and ascorbate peroxidase (APX) activities according to our previously described methods [32]. Briefly, SOD activity was assessed based on its ability to inhibit the photochemical reduction of nitroblue tetrazolium (NBT). CAT activity was determined by monitoring the decomposition of H_2_O_2_ as the decrease in absorbance at 240 nm. POD activity was assayed by measuring the increase in absorbance at 470 nm resulting from guaiacol oxidation. And APX activity was determined by monitoring the decrease in absorbance at 290 nm resulting from ascorbate oxidation.

### 2.4. Transcriptome Sequencing and Data Analysis

Transcriptome sequencing and data analysis were performed as previously described by Wang et al. [27]. RNA-seq libraries were prepared from total RNA extracted from plant tissues with the RNAprep Pure Plant Kit (Tiangen, Beijing, China). RNA quality was assessed by NanoDrop 2000 spectrophotometer (Thermo Fisher Scientific, Waltham, MA, USA) and Agilent 2100 Bioanalyzer (Agilent Technologies, Santa Clara, CA, USA). Following poly(A)+ mRNA enrichment and cDNA synthesis using the Dual-mode mRNA Library Prep Kit (Yeasen Biotechnology, Shanghai, China), libraries were sequenced on an Illumina NovaSeq 6000 platform (Illumina, Inc., San Diego, CA, USA). Raw sequencing data were filtered using fastp (v0.20.0), and the resulting clean reads were aligned to the TAIR10 *Arabidopsis thaliana* genome with HISAT2 (v2.2.1). Read counts were generated using StringTie (v2.0.4), followed by differential expression analysis with DESeq2 (v1.26.0). Multiple testing was controlled using the Benjamini–Hochberg procedure, and genes with FDR < 0.05 and |log2FC| ≥ 1 were defined as DEGs. Functional enrichment of DEGs was evaluated by GO and KEGG analyses using clusterProfiler (v4.4.4).

### 2.5. Quantitative PCR (qPCR) Analysis

Total RNA was extracted from Arabidopsis seedlings using the Total RNA Extraction Kit (R2060, Solarbio, Beijing, China), followed by cDNA synthesis with the HiScript cDNA Synthesis Kit (R312-01, Vazyme, Nanjing, China). Quantitative PCR was conducted on a CFX96 Real-Time PCR System (Bio-Rad, Hercules, CA, USA) using ChamQ SYBR qPCR Master Mix (Q311-02, Vazyme, Nanjing, China) under the manufacturer’s recommended conditions. Relative gene expression was calculated using the 2^−ΔΔCt^ method [32] with *AtUBQ10* as the reference gene. Primer sequences are listed in Supplementary Table S1.

### 2.6. LC-MS and Data Processing

For metabolomic analysis, freeze-dried Arabidopsis tissues were pulverized, and 30 mg of powder was extracted with 1500 μL of −20 °C 70% methanol containing an internal standard. Samples were vortexed for 30 s at 30 min intervals over six cycles and centrifuged at 12,000× *g* for 3 min. The supernatants were membrane-filtered (0.22 μm) and subjected to LC-MS analysis.

Metabolites were profiled using a Vanquish UHPLC-Q Exactive HF-X system (Thermo Fisher Scientific, Waltham, MA, USA) as described previously [33]. Separation was performed on a Waters ACQUITY UPLC HSS T3 column (100 × 2.1 mm, 1.8 μm; Waters Corporation, Milford, MA, USA) at 40 °C using 0.1% formic acid in water/acetonitrile as the mobile phases, with a flow rate of 0.40 mL min^−1^ and a 4 μL injection volume. MS data were acquired in both ESI polarities using data-dependent acquisition over *m*/*z* 84–1250 at a resolution of 35,000. Stepped NCEs of 30, 40, and 50 were used for MS/MS fragmentation, with the top 10 precursor ions selected and a 3 s dynamic exclusion.

Raw data were processed with XCMS for feature detection, alignment, and retention-time correction [34]. Features with >50% missing values were excluded, and signal intensities were normalized using support vector regression. Metabolite annotation was performed using an in-house spectral library, public and predicted databases, and metDNA. Features with identification scores > 0.5 and QC CVs < 0.5 were retained. Positive- and negative-mode datasets were integrated, and differential metabolites were defined by VIP > 1 and |log2FC| ≥ 1.0.

### 2.7. Statistical Analysis

GO enrichment of DEGs was assessed using the Goseq R package (v1.56.0) based on the Wallenius non-central hypergeometric distribution, while GSEA was performed with GSEA software (v4.1.0) to identify coordinated enrichment of predefined gene sets. PCA was conducted using factoextra (v1.0.7). MA and volcano plots were generated with DESeq2 (v1.26.0). OPLS-DA was implemented in ropls (v1.6.2), with model validity assessed by 200 permutation tests. Statistical analyses were performed using SPSS v13.0, with treatment effects tested by one-way ANOVA followed by Tukey’s multiple comparison test. Differences were considered significant at *p* < 0.05.

## 3. Results

### 3.1. Pyrophosphate Alleviates Cd-Induced Growth Inhibition in Plants

**Figure 1:**
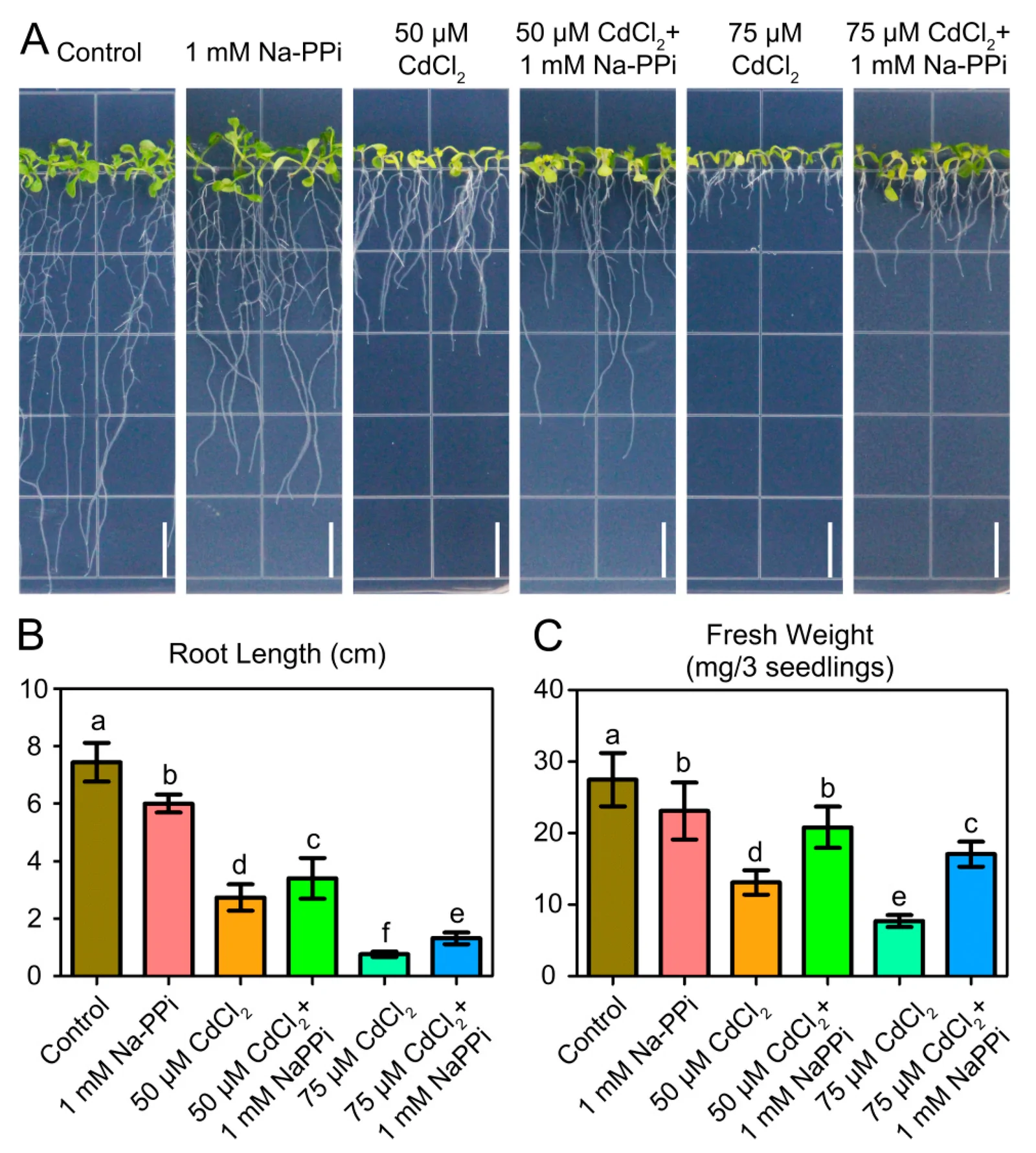
Na-PPi alleviates Cd-induced growth inhibition in Arabidopsis. (**A**) Effects of exogenous application of 1 mM Na-PPi on the growth of 10 DAG Arabidopsis seedlings exposed to 50 μM or 75 μM CdCl_2_; (**B**) quantification of root length, n = 45; (**C**) quantification of fresh weight, n = 15. Data are means ± SD; scale bar = 1 cm. Different lowercase letters above the bars indicate statistically significant differences among treatment groups (*p* < 0.05).

On 1/2 MS medium, 1 mM Na-PPi significantly reduced primary root length (from 7.45 ± 0.68 to 6.00 ± 0.31 cm; Figure 1A,B) and fresh weight (from 27.49 ± 3.74 to 23.10 ± 3.97 mg per 3 seedlings; Figure 1A,C). As expected, exposure to varying concentrations of CdCl_2_ significantly suppressed both root length and fresh weight. However, exogenous application of 1 mM Na-PPi significantly alleviated Cd-induced growth inhibition: under 50 μM CdCl_2_ stress, Na-PPi increased root length and fresh weight by 24.45% (*p* < 0.001) and 58.69% (*p* < 0.001), respectively, compared to CdCl_2_ treatment alone (Figure 1B,C); under 75 μM CdCl_2_ stress, the enhancements were even more pronounced, with root length and fresh weight increasing by 72.12% (*p* < 0.001) and 121.35% (*p* < 0.001), respectively (Figure 1B,C).

A pot experiment was further conducted to evaluate the effect of Na-PPi in alleviating Cd stress in plants. The results showed that Cd stress significantly inhibited the growth of *Lavandula angustifolia*—an aromatic economic crop valued for its medicinal, culinary, ornamental, and ecological properties. Exogenous application of 1 mM Na-PPi significantly mitigated this growth inhibition, as evidenced by increases of 47.74% in plant height and 20.61% in root length (Figure S1A,B), along with 23.89% and 27.59% enhancements in the fresh weights of shoots and roots, respectively (Figure S1A,C), compared to the Cd-stressed control. Notably, under non-Cd-stressed conditions, treatment with 1 mM Na-PPi significantly reduced root length (from 17.53 cm to 15.45 cm) but did not exert a significant effect on root fresh weight (Figure S1B,C).

### 3.2. Effects of Na-PPi on Cd Accumulation, Oxidative Stress, and Antioxidant Enzyme Activities in Cd-Exposed Arabidopsis

**Figure 2:**
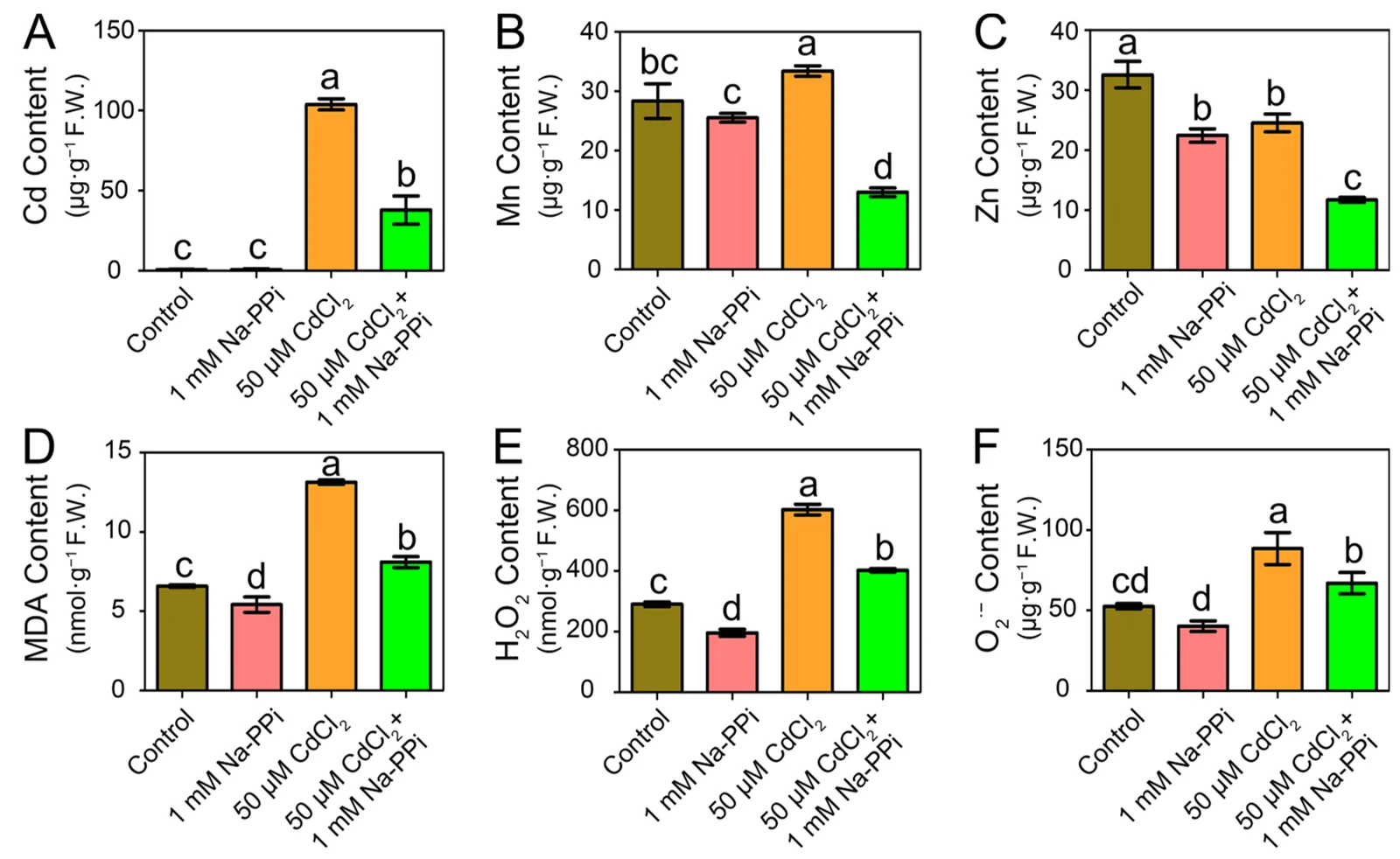
Effects of Na-PPi and CdCl_2_ treatments on metal accumulation and oxidative status in Arabidopsis. (**A**) Cd content; (**B**) Mn content; (**C**) Zn content; (**D**) MDA content; (**E**) H_2_O_2_ content; (**F**) O_2_^·−^ accumulation. Data are means ± SD (n = 5 biological replicates). Different lowercase letters indicate significant differences among treatments.

Further analysis revealed that the concentrations of Cd (Figure 2A), Mn (Figure 2B), and Zn (Figure 2C) in Arabidopsis were all significantly lower in the “50 μM CdCl_2_ + 1 mM Na-PPi” treatment group compared to the “50 μM CdCl_2_” group. Meanwhile, a similar reduction trend was also observed in MDA content (Figure 2D). Moreover, the application of 1 mM Na-PPi significantly reduced the accumulation of H_2_O_2_ (Figure 2E) and O_2_^·−^ (Figure 2F) relative to the 50 μM CdCl_2_ treatment alone. Under 50 μM CdCl_2_ treatment, 1 mM Na-PPi significantly enhanced the activities of SOD (Figure S2A) and APX (Figure S2C), whereas its effects on CAT (Figure S2B) and POD (Figure S2D) activities were relatively limited.

### 3.3. Transcriptome Analysis

**Figure 3:**
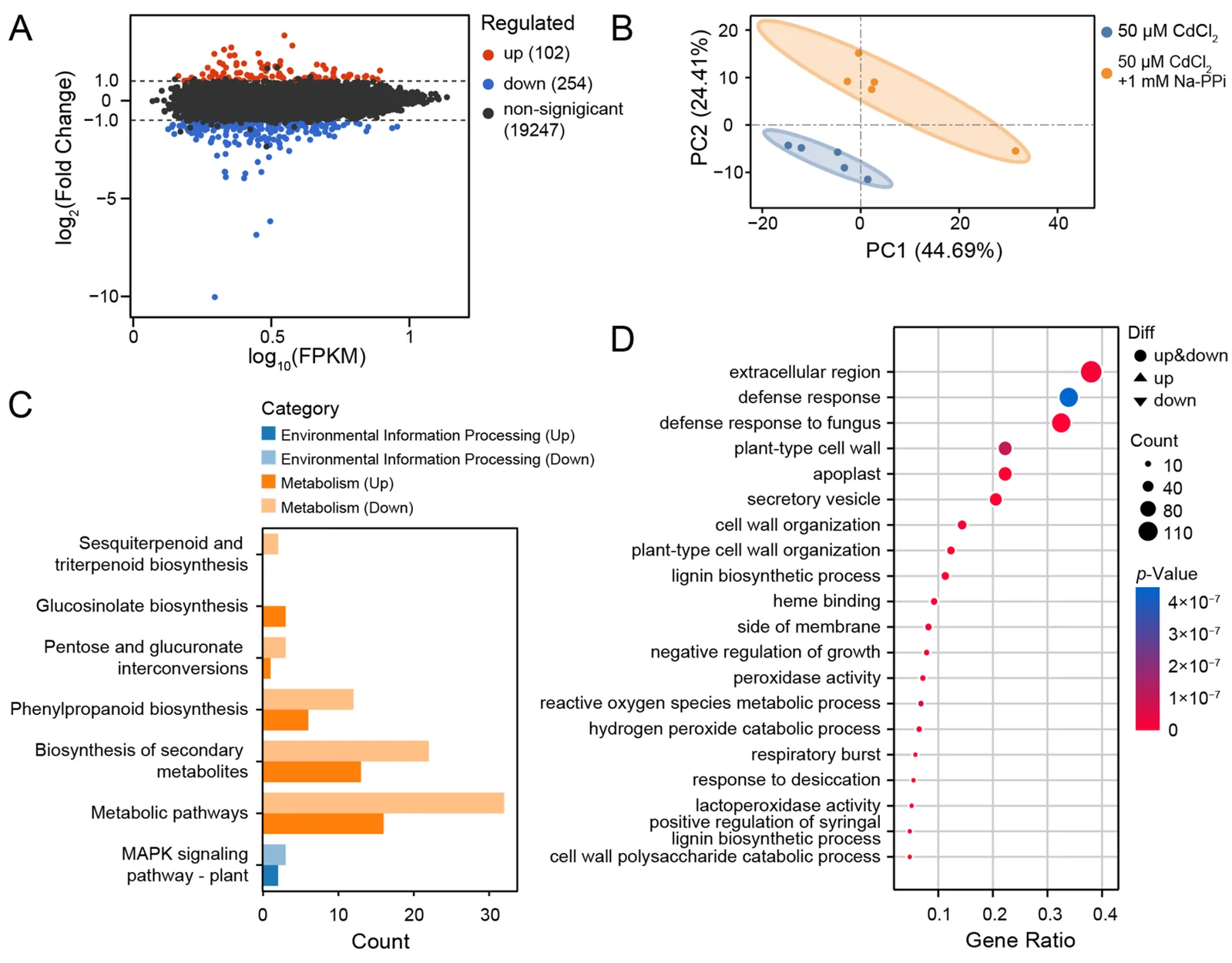
Transcriptomic profiling and functional enrichment analysis of Arabidopsis under CdCl_2_ stress with or without Na-PPi treatment. (**A**) MA plot showing DEGs between the 50 μM CdCl_2_ and 50 μM CdCl_2_ + 1 mM Na-PPi treatments (|log_2_(Fold Change)| ≥ 1, FDR < 0.05). Red: upregulated; blue: downregulated; black: non-significant, n = 5. (**B**) PCA of transcriptome data. (**C**) KEGG pathway enrichment analysis of DEGs. Enriched pathways are ranked by gene count. (**D**) GO biological process enrichment analysis. Bubble size represents gene count; color bar reflects *p* value. Only the top 20 significant terms are shown.

Transcriptome analysis identified a total of 356 DEGs between the “50 μM CdCl_2_” and “50 μM CdCl_2_ + 1 mM Na-PPi” treatment groups, of which 102 were significantly upregulated and 254 were significantly downregulated in response to Na-PPi (Figure 3A). Principal component analysis (PCA) revealed a clear separation between the two groups in global gene expression patterns, with the first principal component (PC1) accounting for 44.69% and the second principal component (PC2) for 24.41% of the total variance (Figure 3B). KEGG pathway enrichment analysis revealed that DEGs modulated by Na-PPi under CdCl_2_ stress were primarily enriched in “Metabolic pathways”, “Biosynthesis of secondary metabolites”, and “Phenylpropanoid biosynthesis” (Figure 3C). GO enrichment analysis showed that these DEGs were significantly enriched in cell wall-related biological processes, including “plant-type cell wall”, “cell wall organization”, “lignin biosynthetic process”, and “cell wall polysaccharide catabolic process” (Figure 3D). Meanwhile, most of these differentially expressed genes involved in cell wall regulation belong to four major families: peroxidases (PER; 14 members), cytochrome P450s (CYP; 6 members), casparian strip membrane domain proteins (CASP; 5 members), and xyloglucan endotransglucosylase/hydrolases (XTH; 5 members) (Figure S3).

Measurements of cell wall components showed that 1 mM Na-PPi significantly reduced cellulose content relative to controls under both 0 and 50 μM CdCl_2_ conditions (Figure S4A). CdCl_2_ stress significantly increased hemicellulose content compared with both the control and the 1 mM Na-PPi treatments (Figure S4B). Meanwhile, the ionic-soluble pectin (ISP) content remained largely unchanged in response to either Na-PPi or CdCl_2_ treatment (Figure S4C). Furthermore, exogenous Na-PPi significantly increased covalently soluble pectin (CSP) content under both the control and CdCl_2_ treatments, as compared with their respective counterparts without Na-PPi (Figure S4D).

### 3.4. Nitrogen Metabolism Is Involved in Na-PPi-Mediated Cd Stress Tolerance in Arabidopsis

**Figure 4:**
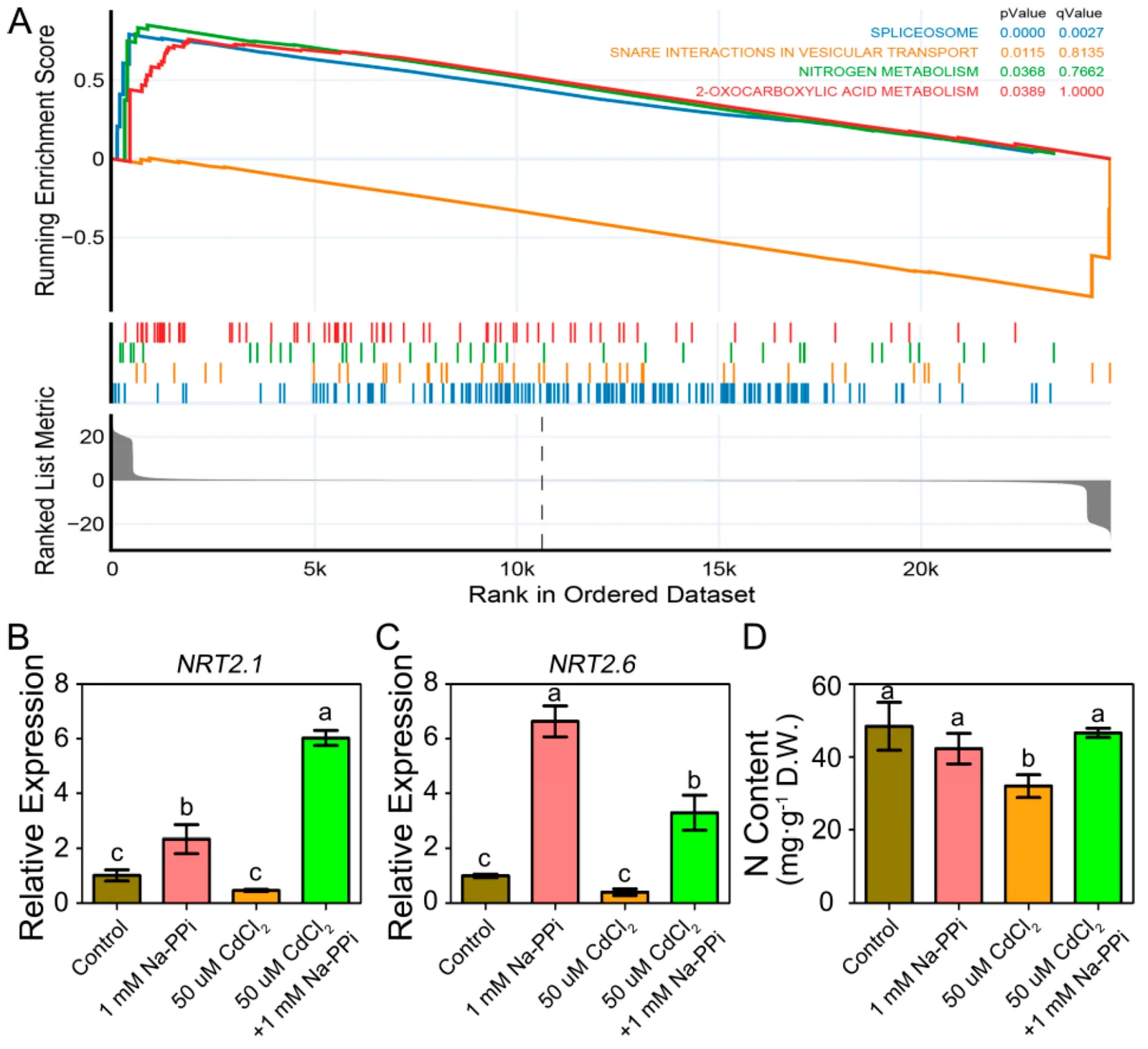
Na-PPi regulates nitrogen metabolism in Arabidopsis under Cd stress. (**A**) GSEA enrichment analysis. (**B**) Relative expression level of the *NRT2.1* gene (n = 3 biological replicates). (**C**) Relative expression level of the *NRT2.6* gene (n = 3 biological replicates). (**D**) Total nitrogen content in Arabidopsis seedlings (n = 5 biological replicates). Data are means ± SD. Different lowercase letters indicate significant differences among treatments.

Gene Set Enrichment Analysis (GSEA) was employed to further evaluate the enrichment status of predefined gene sets between the “50 μM CdCl_2_” treatment group and the “50 μM CdCl_2_ + 1 mM Na-PPi” treatment group. Among the enriched pathways with |NES| > 1.4, only the “Spliceosome” pathway reached statistical significance after multiple-testing correction (*p* < 0.05, q-value < 0.05) (Figure 4A). Additionally, the pathways “SNARE interactions in vesicular transport”, “Nitrogen metabolism”, and “2-Oxocarboxylic acid metabolism” exhibited *p* < 0.05 but q-value > 0.05, suggesting they may possess potential biological relevance and warrant further investigation. In the GSEA-enriched nitrogen metabolism pathway, the DEGs consisted of *NRT2.1* and *NRT2.6*. qPCR analysis showed that, compared with the 1/2 MS control, Na-PPi treatment significantly upregulated the expression of *NRT2.1* and *NRT2.6*, with transcript levels increasing by 2.30-fold and 6.63-fold, respectively (Figure 4B). Under 50 μM CdCl_2_ stress, exogenous Na-PPi similarly and significantly enhanced the expression of *NRT2.1* and *NRT2.6*, reaching 13.38-fold and 8.21-fold of those in the Cd treatment, respectively (Figure 4C). Furthermore, total nitrogen analysis revealed that Cd stress significantly reduced the total nitrogen content of Arabidopsis seedlings, whereas exogenous Na-PPi treatment significantly alleviated the Cd-induced decline in total nitrogen content (Figure 4D).

### 3.5. Metabolomics Analysis

**Figure 5:**
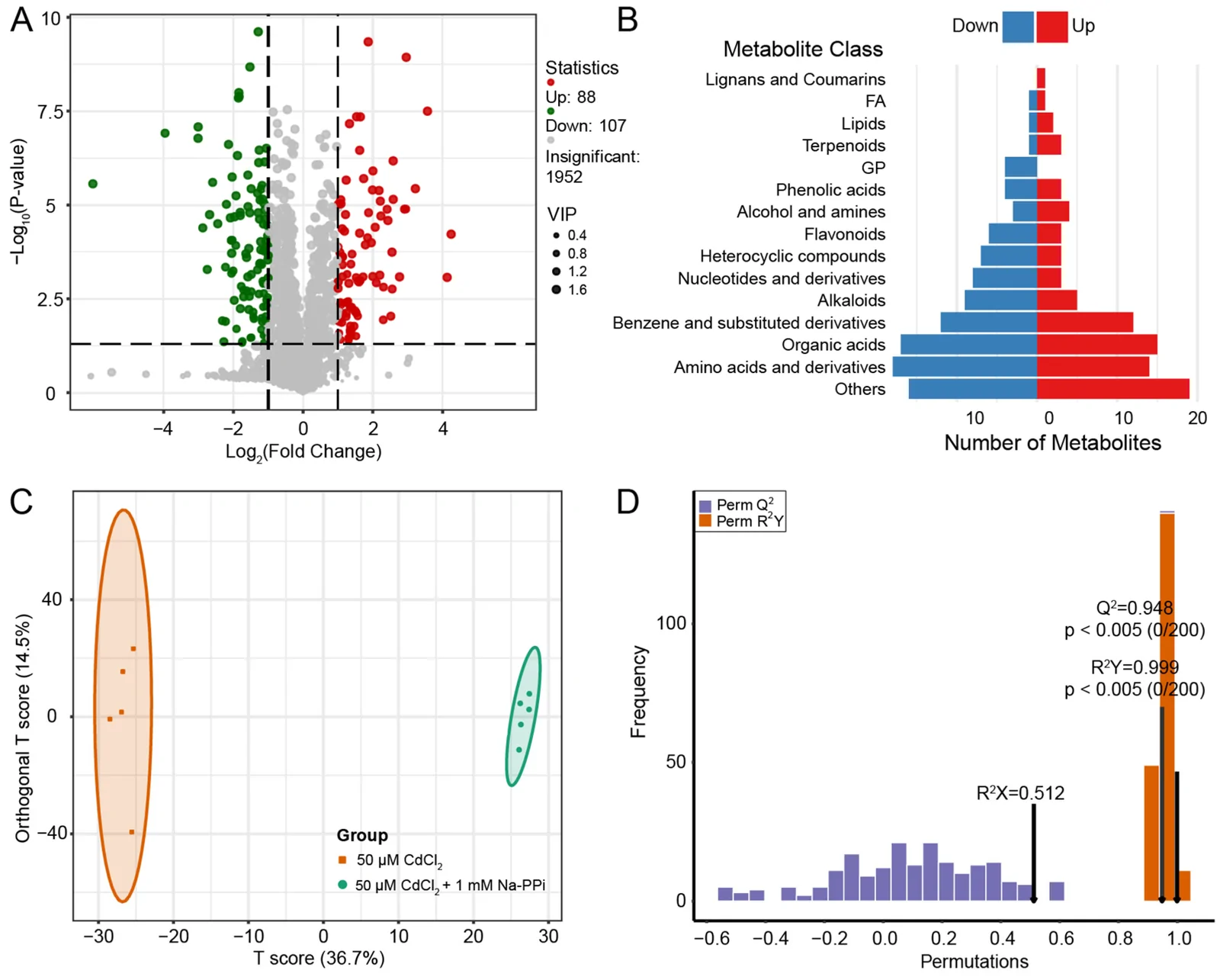
Metabolomic profiling of Arabidopsis reveals Na-PPi-mediated metabolic reprogramming under Cd stress. (**A**) Volcano plot depicting differentially abundant metabolites between the “50 μM CdCl_2_” and “50 μM CdCl_2_ + 1 mM Na-PPi” treatment groups. Significantly upregulated and downregulated metabolites are shown in red and green, respectively. (**B**) Distribution of differentially abundant metabolites across metabolic classes. Red and blue bars represent upregulated and downregulated metabolites, respectively. (**C**) Score plot of the orthogonal partial least squares discriminant analysis (OPLS-DA) model, showing clear separation between the two treatment groups. (**D**) Permutation test (n = 200) assessing the robustness of the OPLS-DA model. The histogram displays the distributions of R^2^Y and Q^2^ values obtained from random permutations. The original model exhibits high R^2^Y and Q^2^ values, with R^2^X = 0.512, confirming its validity and absence of overfitting.

Furthermore, an untargeted metabolomics approach was employed to characterize the metabolic responses of Arabidopsis to CdCl_2_ stress modulated by Na-PPi. A total of 2147 metabolites were identified by LC-MS, among which 88 were significantly upregulated and 107 were significantly downregulated upon Na-PPi treatment (Figure 5A). These differentially abundant metabolites were primarily enriched in metabolic categories including “amino acids and derivatives”, “organic acids”, “benzene and substituted derivatives”, and “alkaloids” (Figure 5B). Meanwhile, these metabolites were significantly enriched in several canonical metabolic pathways, including “Metabolic pathways”, “Biosynthesis of secondary metabolites”, “Biosynthesis of amino acids”, “Purine metabolism”, and “Nucleotide metabolism” (Figure S5). OPLS-DA revealed a clear separation between the CdCl_2_-treated group and the CdCl_2_ + Na-PPi-treated group in the score plot, indicating that Na-PPi substantially reprogrammed the metabolic profile of Arabidopsis under Cd stress (Figure 5C). Permutation testing (n = 200) further validated the robustness of the OPLS-DA model: the original model yielded R^2^Y = 0.990 and Q^2^ = 0.948, both significantly higher than the corresponding values from permuted models (*p* < 0.005), confirming excellent model fit and predictive ability without overfitting (Figure 5D).

### 3.6. LC-MS-Identified Metabolites Typically Responsive to Cd Stress in Arabidopsis

**Figure 6:**
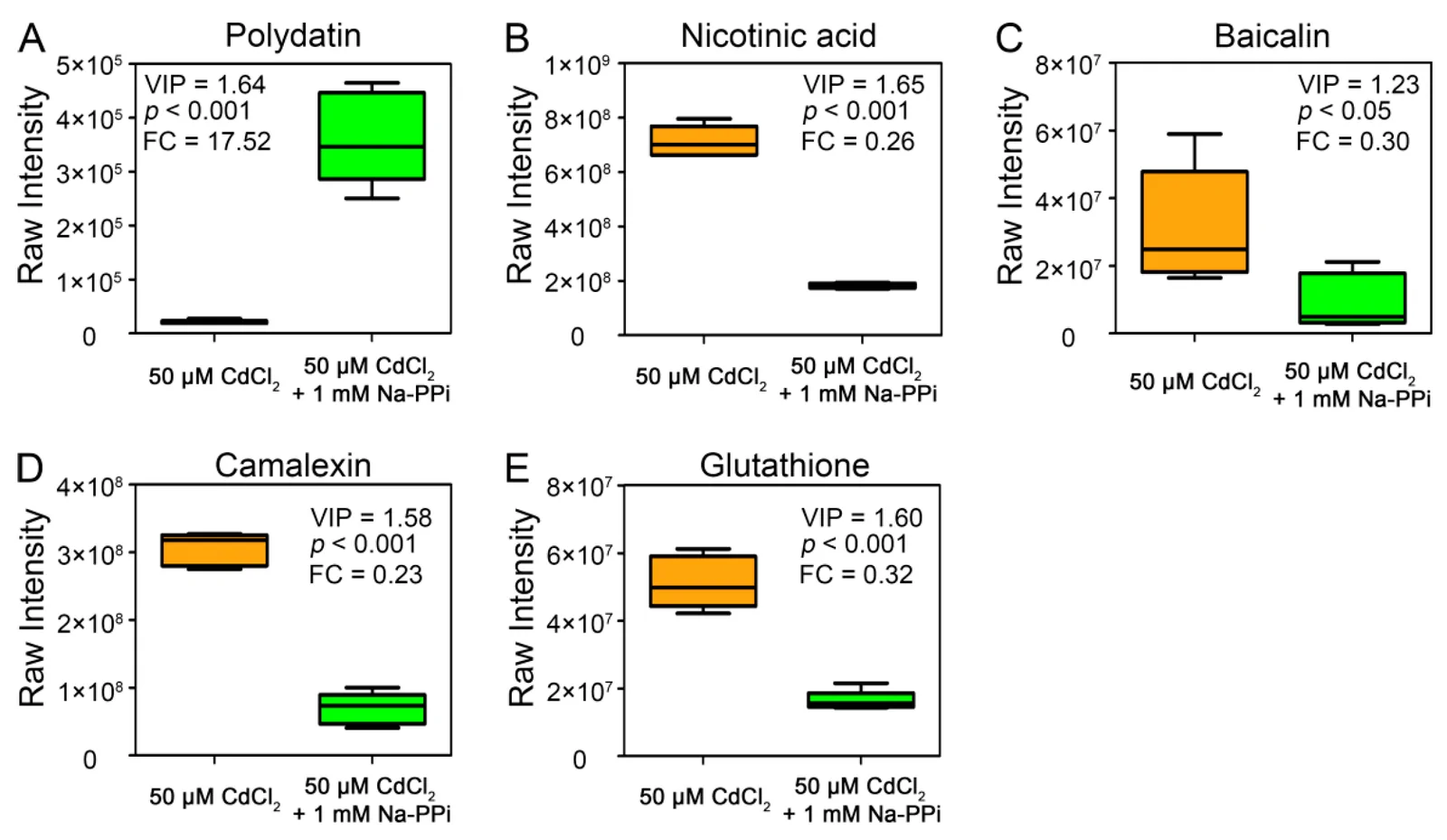
Canonical differential metabolites linked to Cd stress in plants. (**A**) Polydatin; (**B**) Nicotinic acid; (**C**) Baicalin; (**D**) Camalexin; (**E**) Glutathione. The selection criteria indicated in the figure include VIP scores, *p*-values, and fold change (FC) values.

Furthermore, we identified key metabolites associated with plant Cd stress tolerance among the differential metabolites. Compared with 50 μM CdCl_2_ treatment, exogenous application of 1 mM Na-PPi significantly increased the level of polydatin by 17.52-fold (Figure 6A). In contrast, the abundances of nicotinic acid (Figure 6B), baicalin (Figure 6C), camalexin (Figure 6D), and glutathione (Figure 6E) were significantly reduced by 2.85-, 2.33-, 3.35-, and 2.13-fold, respectively.

## 4. Discussion

### 4.1. Does Na-PPi Alleviate Cd Stress in Plants Solely by Reducing Free Cd in the Environment?

Pyrophosphate has long been thought to alleviate Cd-induced phytotoxicity predominantly through its ability to sequester free Cd^2+^ ions in soil by forming insoluble Cd-pyrophosphate precipitates via coordination bonding, thereby decreasing Cd bioavailability [15,35]. Nevertheless, PPi also plays a critical regulatory role in plant growth, development, and stress responses. For instance, loss-of-function mutations in pyrophosphatases disrupt cellular PPi homeostasis, leading to a range of developmental defects including aberrant cotyledon morphology, impaired growth, and even pistil sterility [24,36]. Under hypoxic conditions, ATP synthesis in plants is suppressed, and PPi may serve as an alternative energy source to support cellular metabolism [26]. Furthermore, under P deficiency, plants can hydrolyze PPi to release inorganic phosphate, thereby alleviating the physiological stress caused by phosphorus limitation [37]. The present study demonstrates that exogenous application of 1 mM Na-PPi significantly alleviated the growth inhibition caused by 50 μM CdCl_2_ stress in both Arabidopsis and lavender, and significantly reduced Cd accumulation in Arabidopsis. Previous studies have shown that overexpression of V-PPase hydrolyzes PPi to release energy, thereby enhancing vacuolar compartmentalization of metal ions, mitigating cellular damage caused by heavy metal stress, and ultimately improving plant tolerance to Cd stress [38,39]. However, our previous studies indicated that exogenous application of Na-PPi has only a limited effect on the activity of V-PPase in Arabidopsis [24], indicating that Na-PPi may not alleviate the inhibitory effect of Cd stress on plant growth via the V-PPase pathway. In contrast to the exacerbation of ROS burst in Arabidopsis under salt stress by exogenous Na-PPi [24], the present study demonstrates that Na-PPi treatment significantly reduced the accumulation of MDA and ROS under Cd stress. This protective effect is likely attributable to the decreased Cd accumulation in Arabidopsis and the enhanced activities of SOD and APX following Na-PPi application. Meanwhile, the regulatory effects of Na-PPi on the expression of key genes involved in nitrogen metabolism and associated metabolic profiles under Cd stress (discussed in detail below) suggest that its role in enhancing plant Cd tolerance is multifaceted, encompassing both beneficial effects and potentially adverse or inhibitory components.

### 4.2. Na-PPi-Regulated DEGs Involved in Cell Wall Biological Processes and Nitrogen Metabolism Under Cd Stress

The plant cell wall functions as the primary barrier limiting excessive Cd influx into the cytoplasm and is widely recognized as a key compartment for Cd sequestration [40,41]. Rather than acting as a passive biosorbent, the cell wall constitutes a complex and dynamic architecture in which cellulose microfibrils are intricately embedded within a matrix of polysaccharides, proteins, lignin, and other structural components [42,43]. This matrix is enriched with hemicellulosic polysaccharides (e.g., glucans, heteroglucans, and mannans) and pectic polysaccharides, all of which harbor abundant functional groups such as carboxyl, acylamino, and hydroxyl groups that are capable of establishing electrostatic interactions with heavy metal cations [44,45]. Multiple transcriptomic analyses have also demonstrated that Cd stress induces a dynamic transcriptional response in genes associated with cell wall metabolism [46,47,48]. Moreover, certain heavy metal detoxifying agents have been shown to alleviate Cd-induced phytotoxicity by enhancing Cd immobilization in the cell wall through transcriptional modulation of the phenylpropanoid pathway, polysaccharide biosynthesis, and cell wall biogenesis and remodeling [49,50]. Furthermore, cell wall component analysis showed that, compared with CdCl_2_ treatment alone, exogenous Na-PPi significantly decreased cellulose content but significantly increased CSP content, suggesting a possible reorganization of cell wall components induced by Na-PPi. Notably, although Na-PPi alleviated Cd stress, cellulose content declined, which is consistent with previously reported changes in cellulose content in rice roots under Cd stress with Aeration treatment [51]. Given that CSP plays a critical role in Cd adsorption to cell walls [52], this may represent one of the potential mechanisms underlying the Na-PPi-mediated Cd tolerance in Arabidopsis. As a central component of plant defense, the phenylpropanoid biosynthetic pathway terminates in the production of lignin, which reinforces cellular structures and provides a critical first line of defense against abiotic stresses [49,53]. In this study, exogenous application of Na-PPi significantly enriched multiple DEGs involved in phenylpropanoid biosynthesis and cell wall metabolism. However, it remains unclear whether these DEGs are a direct consequence of Na-PPi treatment or an indirect effect resulting from the Na-PPi-mediated reduction in Cd content.

Nitrogen is an essential macronutrient indispensable for plant growth and productivity. Cd stress could significantly reduce plant N content by inhibiting the activities of nitrate reductase and nitrite reductase, which are critical for nitrate assimilation [3,54]. Furthermore, at the molecular level, nitrate uptake is mediated by members of the NRT1 and NRT2 transporter families, which function as low-affinity and high-affinity NO_3_^−^ transporters, respectively. Meanwhile, previous studies have demonstrated that cadmium stress significantly suppresses the expression of *NRT2.1*, *NRT2.2*, and *NRT2.4* genes [55]. Guan et al. proposed that NRT2.1 functions as the principal mediator of nitrate uptake under Cd stress in low-NO_3_^−^ conditions, contributing approximately 50% to total nitrate acquisition [56]. In this study, Cd stress significantly suppressed the expression of *NRT2.1*; however, Na-PPi treatment effectively alleviated this repression, elevating *NRT2.1* transcript levels to approximately 13.38-fold those observed under Cd-stress conditions. This upregulation may represent a key molecular mechanism underlying the enhancement of plant Cd tolerance by Na-PPi at the plant level. Nevertheless, further genetic experiments are required to determine whether *NRT2.1* plays a decisive role in Na-PPi-mediated Cd stress tolerance. The expression of *NRT2.6* is minimally affected by nitrogen deficiency but is closely associated with the accumulation of ROS induced by biotic and abiotic stresses [57,58]. In this study, the expression of *NRT2.6* was also induced by Na-PPi treatment, suggesting that Na-PPi may represent a novel regulatory factor distinct from ROS.

### 4.3. Differential Metabolites Mediated by Na-PPi Under Cd Stress

Metabolomics is a powerful tool for analyzing plant responses to Cd stress. Zhang et al. demonstrated that Cd stress significantly altered the metabolite profile in the roots of *Codonopsis pilosula*, with differentially expressed metabolites primarily classified into categories including amino acids, lipids, carbohydrates, and other secondary metabolites [59]. Furthermore, previous studies have demonstrated that exogenous application of glutathione (GSH) can effectively alleviate Cd-induced stress in plants; metabolomic analyses further revealed that differential metabolites induced by GSH treatment are predominantly enriched in metabolic pathways associated with organic acids and derivatives and benzenoids [60], which is consistent with those observed in the present study. In addition, amino acids, organic acids, and peptides have been identified as key ligands that facilitate Cd precipitation through chelation [61]. Notably, differential metabolites, including several amino acid and derivative compounds, were found to play a critical role in mitigating Cd toxicity under boron supplementation [62].

Among the differential metabolites, several associated with Cd stress have also drawn our attention. Polydatin is a glycosylated derivative of resveratrol formed at the C3-hydroxyl (C3-OH) position and serves as a key bioactive constituent in various traditional Chinese medicinal herbs [63,64]. Previous studies have shown that an appropriate concentration of Cd significantly enhances polydatin content in *T. hemsleyanum* [65]. Moreover, polydatin can effectively alleviate Cd-induced oxidative stress in animal cells by enhancing the activity of antioxidant systems [66,67]. This study demonstrates that Na-PPi significantly enhances the accumulation of polydatin in Arabidopsis under Cd stress; however, whether polydatin is involved in the scavenging of Cd-induced ROS in plants remains to be elucidated. Furthermore, baicalin, a flavonoid biosynthesized via the flavonoid pathway, ameliorates Cd-induced hepatotoxicity by mitigating Cd-triggered cytotoxicity, oxidative stress, and histopathological alterations in liver tissue [68,69]. Under Cd stress, nicotinic acid has been demonstrated to activate defense-associated metabolic pathways in plant cells, primarily by upregulating glutathione biosynthesis [70]. Camalexin is an indolic secondary metabolite that serves as the primary phytoalexin in Arabidopsis [71]. Its biosynthesis is upregulated in response to various exogenous stressors, including pathogen attack, herbicides, and heavy metal ions, with ROS playing an essential role in triggering this biosynthetic pathway [72,73]. Glutathione functions not only as an antioxidant molecule for ROS detoxification but also as a precursor for phytochelatin synthesis involved in Cd sequestration [28]. In this study, Na-PPi alleviated Cd toxicity, thereby reducing the metabolic demand for stress-associated compounds such as glutathione and camalexin. The decreased abundance of these metabolites may indicate a transition from an activated defense state toward metabolic homeostasis rather than impaired antioxidant capacity.

## 5. Conclusions

In this study, we revealed that Na-PPi functions as an effective regulator of plant Cd tolerance rather than merely a Cd immobilization agent. Exogenous Na-PPi application enhanced plant performance under Cd stress by reducing Cd accumulation, maintaining redox homeostasis, and promoting nutrient acquisition. Integrated transcriptomic and metabolomic analyses demonstrated that Na-PPi-mediated Cd tolerance involved coordinated regulation of cell wall remodeling, phenylpropanoid metabolism, and stress-associated metabolic pathways. The activation of nitrate transporter genes *NRT2.1* and *NRT2.6* highlighted a previously unrecognized role of pyrophosphate in improving nitrogen nutritional status during Cd stress. Meanwhile, metabolic reprogramming induced by Na-PPi suggested a transition from stress-responsive defense activation toward metabolic homeostasis after Cd toxicity alleviation. Collectively, our findings expand the current understanding of pyrophosphate functions in plant heavy metal tolerance and provide a mechanistic framework linking pyrophosphate application with Cd detoxification, nutrient regulation, and metabolic adaptation. Future research should focus on validating the effectiveness of pyrophosphate-based strategies under field conditions for sustainable remediation of Cd-contaminated soils.

## Data Availability

The original contributions presented in this study are included in the article/Supplementary Material. Further inquiries can be directed to the corresponding authors.

## Supplementary Materials

The following supporting information can be downloaded at https://www.mdpi.com/article/10.3390/plants15182864/s1: Figure S1: Effects of Na-PPi on the growth of *Lavandula angustifolia* under Cd stress. Figure S2: Effects of Na-PPi and CdCl_2_ treatments on the activities of key antioxidant enzymes in Arabidopsis. Figure S3: The heatmap displays the DEGs involved in cell wall regulation within GO-enriched pathways, with the expression level changes quantified by the color bar on the right, expressed as log_2_(Fold Change). Figure S4: Determination of cell wall component contents. Figure S5: KEGG classification of the differential metabolites identified between the “50 μM CdCl_2_” and “50 μM CdCl_2_ + 1 mM Na-PPi” treatment groups. Table S1: Primer sequences used for qPCR analysis.
