# Supplementary material for: Physiological, Transcriptional, and Metabolic Responses Associated with Pyrophosphate-Mediated Alleviation of Cadmium Stress in Arabidopsis
Source: Plants (Basel). 2026 Sep 19;15(18):2864. doi: 10.3390/plants15182864 (PMC13610396; doi:10.3390/plants15182864)
Supplement: Supplementary file 1 [file plants-15-02864-s001.zip › plants-4490399-supplementary.pdf]

Title page:

Mechanisms of Sodium Pyrophosphate in Alleviating Cadmium Stress in Plants

Authors: Yang Yang[1,2]; Lu Wang[3]; Pengpeng He[1,2]; Jin Wang[1,2]; Chunqiao Wang[1,2]; Chao Wang[1,2*]; Ziwei Jiao[1,2*].

1 College of Biological Sciences and Technology, Yili Normal University, Yining 835000, China

2 Xinjiang Key Laboratory of Lavender Resource Protection and Utilization, Yili Normal University, Yining 835000, China

3 College of Landscape and Ecological Engineering, Hebei University of Engineering, Handan 056038, China

* Correspondence: Chao Wang, cwang@lzu.edu.cn; Ziwei Jiao, jiaoziwei123@163.com

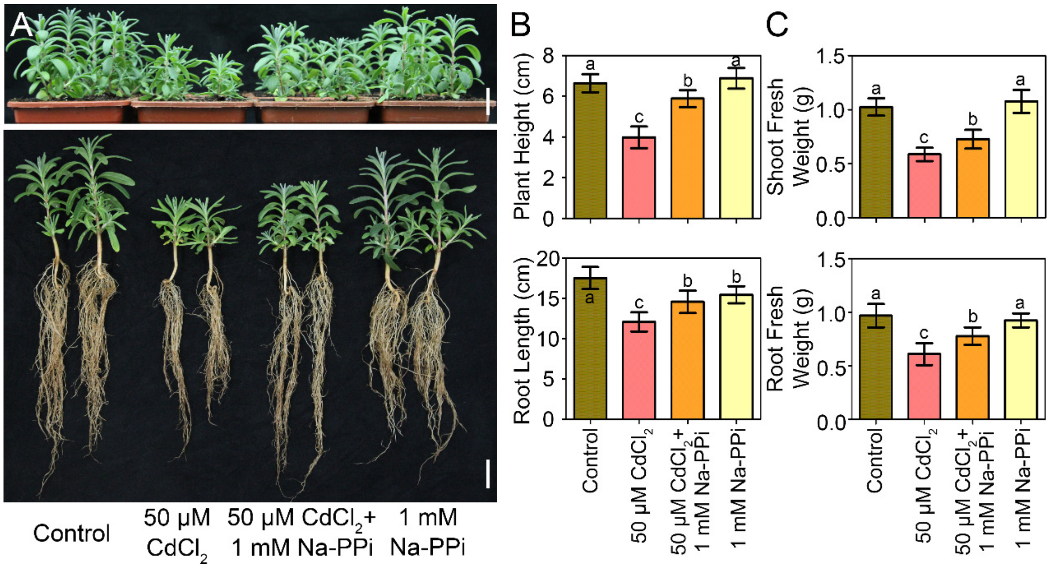

Figure S1 Effects of Na-PPi on the growth of *Lavandula angustifolia* under Cd stress. (A) Growth of lavender plants treated with 1 mM Na-PPi under normal conditions or in the presence of 50 µM $CdCl_2$; (B) quantification of plant height and root length under different growth conditions; (C) quantification of shoot and root fresh weights under different growth conditions. Different lowercase letters above the bars indicate statistically significant differences among treatment groups.

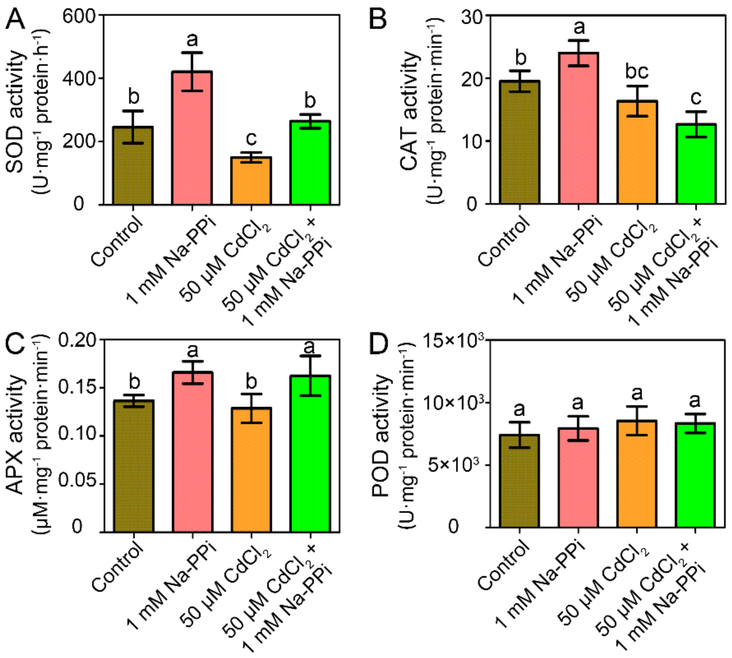

Figure S2 Effects of Na-PPi and $CdCl_2$ treatments on the activities of key antioxidant enzymes in Arabidopsis. (A) SOD activity; (B) CAT activity; (C) APX activity; (D) POD activity. Data are means ± SD (n = 5 biological replicates). Different lowercase letters indicate significant differences among treatments.

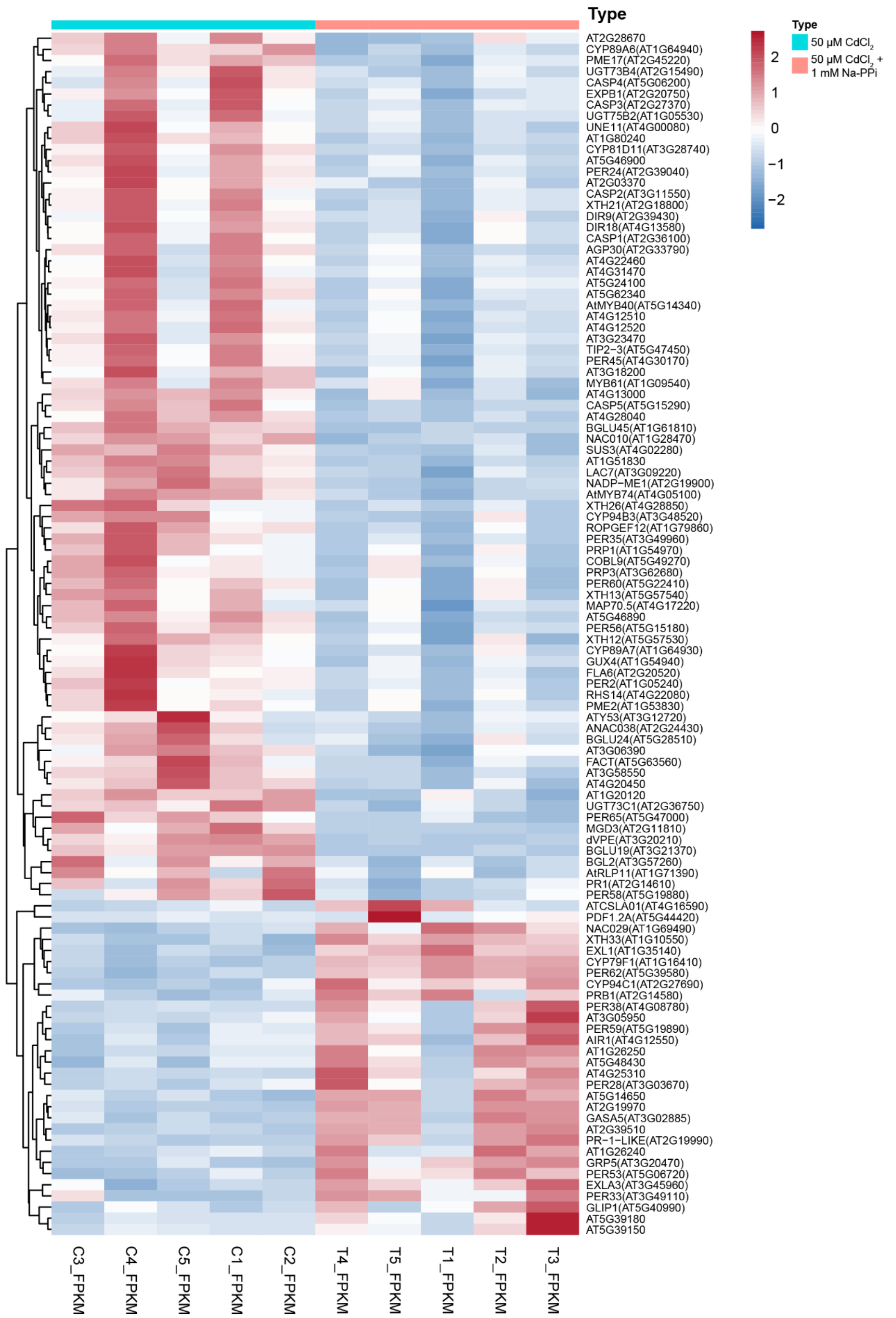

Figure S3 The heatmap displays the DEGs involved in cell wall regulation within GO-enriched pathways, with the expression level changes quantified by the color bar on the right, expressed as $\log_2$(Fold Change).

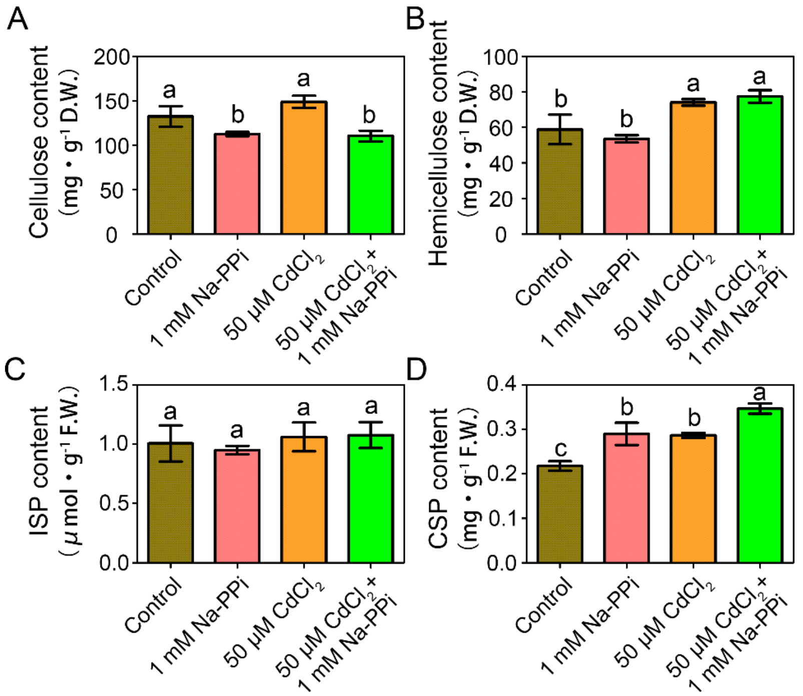

Figure S4 Determination of cell wall component contents. (A) Cellulose content; (B) hemicellulose content; (C) ionic-soluble pectin (ISP) content; (D) covalently-soluble pectin (CSP) content. Different letters indicate significant differences among treatments ($p < 0.05$). Bars represent the mean ± SD; n = 3 biological replicates.

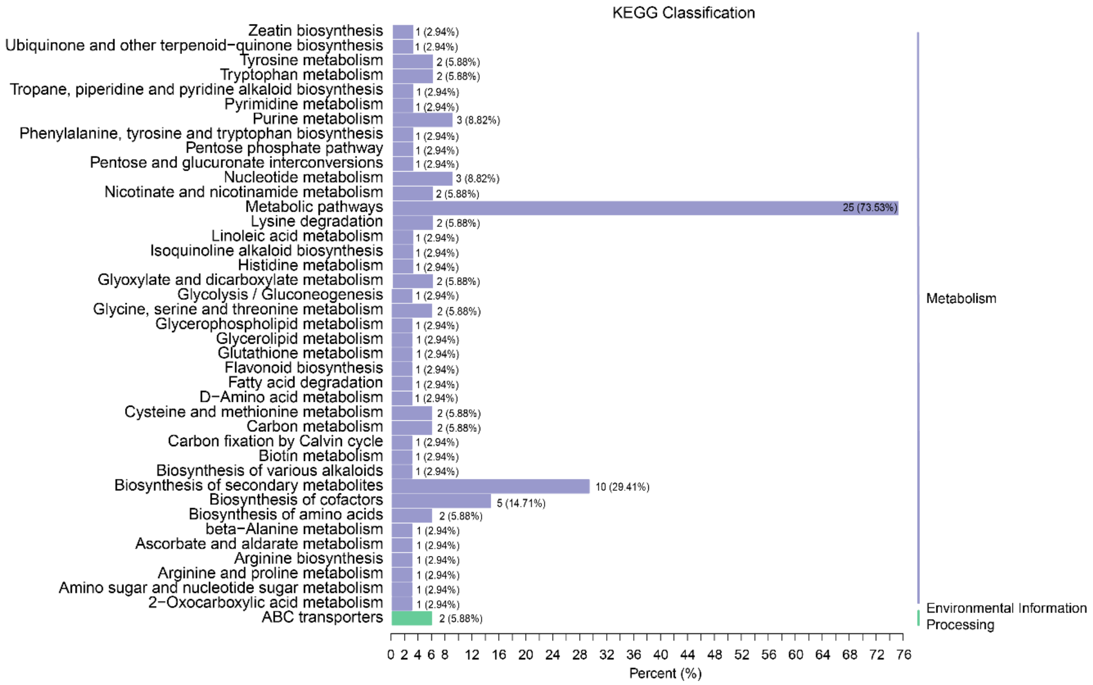

Figure S5 KEGG classification of the differential metabolites identified between the "50 μM $CdCl_2$" and "50 μM $CdCl_2$ + 1 mM Na-PPi" treatment groups.

Table S1. Primer sequences used for qPCR analysis.

Genes	Primer sequences (5'→3')
NRT2.1	F: CTATGGTGCTCTTCTCTATGGG
	R: TTAAACCCGAGATGATGCCTAG
NRT2.6	F: GTTTTCTCCATGTCGTTCGTAG
	R: ACATAACACTCGTCCAGTACTG
UBQ10	F: AATTGGAGGATGGTCGTACTTT
	R: CAAAGTCTTGACGAAGATCTGC
